# Targeted sequencing of the Paget's disease associated 14q32 locus identifies several missense coding variants in *RIN3* that predispose to Paget's disease of bone

**DOI:** 10.1093/hmg/ddv068

**Published:** 2015-02-20

**Authors:** Mahéva Vallet, Dinesh C. Soares, Sachin Wani, Antonia Sophocleous, Jon Warner, Donald M. Salter, Stuart H. Ralston, Omar M.E. Albagha

**Affiliations:** 1 Rheumatology and Bone Disease Section, Centre for Genomic & Experimental Medicine; 2 MRC Human Genetics Unit and Centre for Genomic & Experimental Medicine and; 3 South East Scotland Clinical Genetics Service, Centre for Genomic & Experimental Medicine, Institute of Genetics and Molecular Medicine, Western General Hospital, University of Edinburgh, Edinburgh EH4 2XU, UK

## Abstract

Paget's disease of bone (PDB) is a common disorder with a strong genetic component characterized by increased but disorganized bone remodelling. Previous genome-wide association studies identified a locus on chromosome 14q32 tagged by rs10498635 which was significantly associated with susceptibility to PDB in several European populations. Here we conducted fine-mapping and targeted sequencing of the candidate locus to identify possible functional variants. Imputation in 741 PDB patients and 2699 controls confirmed that the association was confined to a 60 kb region in the *RIN3* gene and conditional analysis adjusting for rs10498635 identified no new independent signals. Sequencing of the *RIN3* gene identified a common missense variant (p.R279C) that was strongly associated with the disease (OR = 0.64; *P* = 1.4 × 10^−9^), and was in strong linkage disequilibrium with rs10498635. A further 13 rare missense variants were identified, seven of which were novel and detected only in PDB cases. When combined, these rare variants were over-represented in cases compared with controls (OR = 3.72; *P* = 8.9 × 10^−10^). Most rare variants were located in a region that encodes a proline-rich, intrinsically disordered domain of the protein and many were predicted to be pathogenic. RIN3 was expressed in bone tissue and its expression level was ∼10-fold higher in osteoclasts compared with osteoblasts. We conclude that susceptibility to PDB at the 14q32 locus is mediated by a combination of common and rare coding variants in *RIN3* and suggest that RIN3 may contribute to PDB susceptibility by affecting osteoclast function.

## Introduction

Paget's disease of bone (PDB) is a common skeletal disorder that affects up to 2% of individuals above the age of 55 in the UK and other populations with founders of European descent (1,2). Genetic factors play an important role in the pathogenesis of Paget's disease. Between 15–30% of patients have a positive family history of the disease and in these families the disease shows an autosomal dominant mode of inheritance with incomplete penetrance (3–6). Mutations have so far been identified in the *SQSTM1* gene as a cause of the disease (7,8) and these occur in up to 40% of patients with familial PDB and up to 10% of those without a family history of the condition. Genome-wide association studies (GWAS) have identified seven loci with robust evidence of association with PDB (9,10). One of these loci, tagged by rs10498635 which is situated on chromosome 14q32.12, was strongly associated with PDB in several European populations with a *P*-value of 2.55 × 10^−11^ and an odds ratio of 1.44 (95% confidence interval 1.29–1.60) for the associated single-nucleotide polymorphism (SNP). The region of strongest association is flanked by two recombination hotspots and contains only the *RIN3* gene that encodes the Ras and Rab interactor protein 3 (11). RIN3 belongs to a family of three proteins that play a role in endocytosis, vesicular trafficking and signal transduction by acting as guanine exchange factors (GEFs) for small GTPases. In particular, RIN3 has been shown to act as a GEF for the Rab5 family of proteins including Rab5 itself and Rab31 (12,13). The role of RIN3 in bone metabolism has not specifically been studied, although it is known that Rab proteins play a role in regulating osteoclast function through effects on vesicular trafficking (14). The aim of this study was to conduct fine-mapping of the *RIN3* locus in order to identify possible functional variants that predispose to PDB.

## Results

### Imputation and association analysis

**Figure 1.**
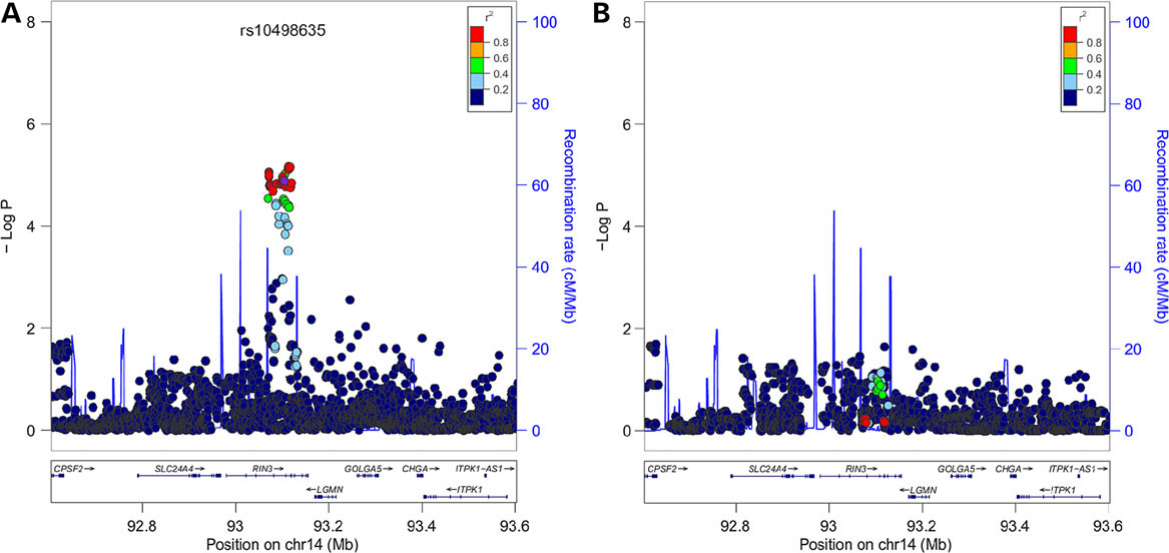
Regional association plots of the chr14q32 region showing the chromosomal position (based on NCBI human genome build 37) of SNPs plotted against −log10 *P*-values obtained from genotyped and imputed SNPs from 741 PDB cases and 2699 controls. SNPs are colour coded according to the extent of LD with rs10498635 (represented as a purple dot) which showed genome-wide significance association in previous GWAS (9). Data before (**A**) and after (**B**) correction for rs10498635. The estimated recombination rates (cM/Mb) from HapMap CEU release 22 are shown as light blue lines, and the blue arrows represent known genes in each region.

The chromosome 14q32 top-hit SNP rs10498635 identified by GWAS (9) is located in intron 4 of the *RIN3* gene and it is not predicted to have any functional effects. It is likely that this SNP is marking for another functional variant in the region. In order to refine the association signals in this region, we carried out imputation using the 1000 genomes data as reference in 741 PDB patients and 2699 controls that were included in our previous genome-wide association study (9). This analysis confirmed that the area of strongest association was limited to a 60 kb region bounded by two recombination hotspots between exons 3 and 8 of *RIN3*. There was a consistent association signal of about 1 × 10^−5^ for common imputed variants surrounding the rs10498635 SNP (Fig. 1A). Some SNPs located outside this region showed nominal association and to test for secondary signals in this locus, we performed conditional analysis by adjusting for rs10498635, but results showed that rs10498635 is the only independent signal from this locus (Fig. 1B).

### Resequencing of RIN3

We next investigated the possibility that previously unrecognized variants within *RIN3* might be responsible for the association observed by conducting deep-sequencing of the 14q32 locus. This included a 210 kb region containing the entire *RIN3* gene and about 20 kb of flanking upstream and downstream sequences using next-generation sequencing (see Materials and Methods). This was carried out in 121 patients with PDB and 49 unaffected controls from the UK. After quality control, we detected 1272 genetic variants of which 1063 were single-nucleotide variants (SNV) and the remaining were indels. We detected 10 missense SNVs in the *RIN3* gene of which four were novel and not reported in public databases including dbSNP, 1000 Genome (www.1000genomes.org/) and NHLBI exome sequencing project (http://evs.gs.washington.edu/EVS/) (Supplementary Material, Table S1). Of the 10 detected missense variants, seven were rare variants (MAF < 1% in 1000 Genomes) that were present only in cases but not in our controls, of which four were not detected in public databases. Additionally, three common missense variants were detected of which one variant (p.R279C) showed a significant association with PDB in this group of cases and controls (*P* = 7.6 × 10^−4^). Non-coding variants were annotated using Ensembl and ENCODE databases to predict their functional significance but only two variants were located within predicted regulatory motifs (Supplementary Material, Table S1); a rare variant detected in the promoter region is predicted to disrupt a transcription factor MafG motif (V-maf; musculoaponeurotic fibrosarcoma oncogene homologue G). This is a member of the small Maf family of proteins that act as transcriptional activators or repressors depending on their interacting partner. Another rare variant was identified in the 5′UTR region, located within a binding site of TAF1 transcription factor as reported by the ENCODE database in three different cell lines (lymphoblastoid, human embryonic stem cells and neuroblastoma). TAF1 (also known as TATA box binding protein associated factor) is the largest component of the RNA polymerase IID (TFIID) transcription factor complex.

We then extended the DNA sequencing by screening an additional 125 PDB cases focusing on the promoter/5′UTR and coding region of the *RIN3* gene using the Sanger DNA sequencing approach (see Materials and Methods). A summary of missense and possible regulatory variants identified from this procedure are shown in Supplementary Material, Table S2. We detected a further six missense rare variants of which three were novel.

**Figure 2.**
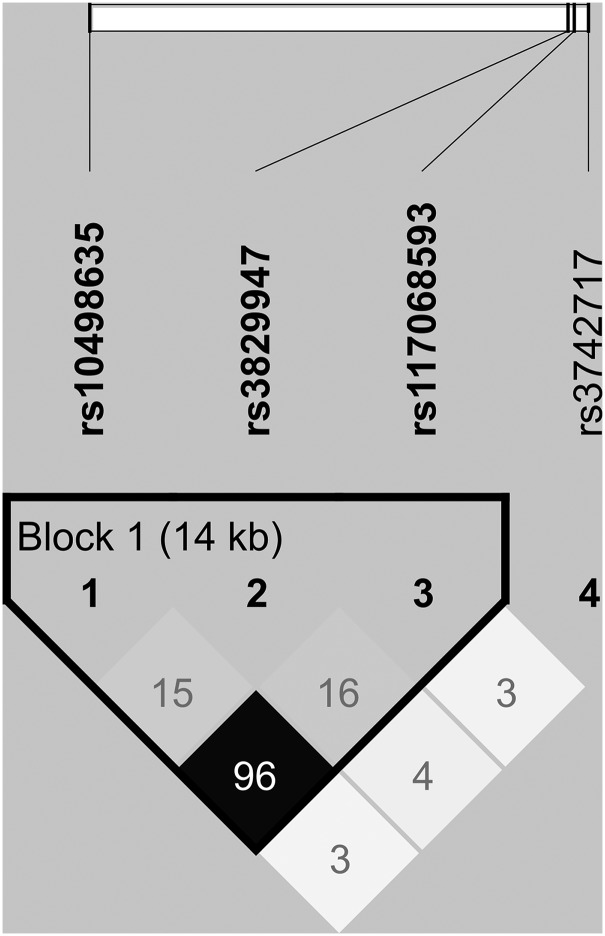
Linkage disequilibrium (LD) plot of common missense coding variants detected by DNA sequencing of *RIN3* and the top GWAS SNP rs10498635.

We next combined variants identified from next-generation sequencing and Sanger sequencing and tested their association with the disease using data from 1000 genomes and NHLBI as controls. A summary of all missense and possible regulatory variants are shown in Table 1. There was a highly significant association between the p.R279C variant (rs117068593) and PDB in the sequenced samples compared with 1000 Genome controls (OR = 0.60; 95% CI = 0.43–0.84; *P* = 3.1 × 10^−3^) and when compared with NHLBI controls (OR = 0.50; 95% CI = 0.38–0.67; *P* = 2.0 × 10^−6^). The p.R279C variant was also associated with PDB in our GWAS cohort of 741 cases and 2699 controls (OR = 0.68; 95% CI = 0.58–0.81; imputed *P* = 5.7 × 10^−6^). When data were combined from all cases and controls, this variant showed strong evidence of association with the disease at a genome-wide level (OR = 0.64; 95% CI = 0.55–0.74; *P* = 1.4 × 10^−9^). The p.R279C variant was found to be in strong linkage disequilibrium (LD) with the top GWAS hit rs10498635 (*r*^2^ = 0.96, D′ = 0.98; Fig. 2). Haplotype analysis in the GWAS cohort identified only two haplotypes (rs10498635C–rs117068593C; frequency in controls = 81.2%, rs10498635T–rs117068593T; frequency = 18.2%, other haplotypes frequency <0.2%). The risk haplotype rs10498635C–rs117068593C was over-represented in the GWAS cases (86.4%) compared with controls (81.2%; OR = 1.48; 95% CI = 1.25–1.74; *P* = 2.8 × 10^−6^). The C allele of R279C was more common in familial cases (*n* = 9; 17.3%) compared with that observed in sporadic cases (*n* = 45; 10.3%) but this was not statistically significant (*P* = 0.12). The other two common coding SNPs (p.H215R and p.T425M) were detected but these did not show a consistent association with the disease in the sequenced samples (Table 1) or in the GWAS cohort of 741 cases and 2699 controls (*P* > 0.09). Additionally, these two SNPs showed no evidence of association with the disease at a genome-wide level in the combined data set (*P* > 1 × 10^−4^) and no LD was found between these two SNPs and the top GWAS hit rs10498635 (*r*^2^ < 0.15; Fig. 2).

**Table 1.** Summary of all missense and possible regulatory variants identified in *RIN3*

Variant ID	Position (hg19)	Reference allele	Sample allele	Gene region	Protein variant	AF cases^a^ (%)	AF 1000G^b^ (%)	*P*-value^c^	AF NHLBI^d^ *N* (%)	*P*-value^e^	Functional prediction score^f^
N/A^g^	92 979 351	A	G	Promoter	—	2/492 (0.41)	0 (0.0)	0.15	—	—	NFE2L1/MafG
rs368389701^h^	92 980 256	C	A	5′UTR	—	1/492 (0.20)	0 (0.0)	0.39	—	—	TFBS (TAF1; POLR2A)
N/A^g^	93 081 806	C	T	Exon 4	p.A141V	1/492 (0.20)	0 (0.0)	0.39	0 (0.0)	0.05	5
rs3829947^g,h^	93 118 038	A	G	Exon 6	p.H215R	257/492 (52.2)	427 (56.3)	0.15	4902 (57.0)	0.04	0
N/A^g^	93 118 085	C	T	Exon 6	p.R231C	1/492 (0.20)	0 (0.0)	0.39	0 (0.0)	0.05	4
rs147329151^g^	93 118 145	C	A	Exon 6	p.Q251K	2/492 (0.41)	0 (0.0)	0.15	2 (0.02)	0.02	1
rs117068593^g,h^	93 118 229	C	T	Exon 6	p.R279C	54/492 (10.97)	129 (17.0)	3.1 × 10^−3^	1687 (19.6)	2.0 × 10^−6^	4
N/A^g^	93 118 260	T	C	Exon 6	p.L289P	1/492 (0.20)	0 (0.0)	0.39	0 (0.0)	0.05	1
N/A^g^	93 118 268	T	C	Exon 6	p.C292R	1/492 (0.20)	0 (0.0)	0.39	0 (0.0)	0.05	2
N/A^g^	93 118 274	C	T	Exon 6	p.P294S	1/492 (0.20)	0 (0.0)	0.39	0 (0.0)	0.05	1
N/A^h^	93 118 310	G	A	Exon 6	p.A306T	1/492 (0.20)	0 (0.0)	0.39	0 (0.0)	0.05	0
rs201271121^g^	93 118 550	C	T	Exon 6	p.P386S	1/492 (0.20)	0 (0.0)	0.39	0 (0.0)	0.05	4
rs3742717^g,h^	93 118 668	C	T	Exon 6	p.T425M	77/492 (15.65)	165 (21.8)	7.5 × 10^−3^	1458 (16.9)	0.45	1
rs74074811^g^	93 118 674	G	A	Exon 6	p.R427Q	2/492 (0.41)	0 (0.0)	0.15	10 (0.12)	0.11	0
rs74074812^g^	93 118 823	C	T	Exon 6	p.P477S	1/492 (0.20)	0 (0.0)	0.39	2 (0.02)	0.14	3
rs12434929^g,h^	93 119 232	G	C	Exon 6	p.G613A	4/492 (0.81)	7 (0.92)	0.24	57 (0.66)	0.19	1
rs145292991^g^	93 125 790	G	A	Exon 7	p.D771N	1/492 (0.20)	0 (0.0)	0.39	2 (0.02)	0.14	3
rs147042536^g^	93 142 861	T	C	Exon 8	p.Y793H	5/492 (1.02)	5 (0.66)	0.19	54 (0.63)	0.11	6

^a^Allele frequency (AF) shown as number of alleles observed/total number of alleles.

^b^Allele frequency in European subjects from 1000 Genomes (*n* = 379).

^c^*P*-value from testing sequenced cases (*n* = 246) and European subjects from 1000 Genome.

^d^Allele frequency in European-American subjects in NHLBI data set (*n* = 4300).

^e^*P*-value from testing sequenced cases (*n* = 246) and European-American subjects from NHLBI data set.

^f^For missense variants, as assessed by SIFT, PolyPhen-2, Condel, MutationTaster, GERP conservation score and Grantham score (see Materials and Methods).

^g^Variant detected in sporadic cases.

^h^Variant detected in familial cases.

Several rare variants (MAF < 1%) were also identified (Table 1) of which two were detected in familial cases, 12 in sporadic cases and one was detected in both groups. Most rare variants were located within exon 6 (10/15) in close proximity to the p.R279C common variant. Genotyping additional family members for variants detected in familial cases revealed that the G613A was transmitted from an affected father (G/A) to an affected daughter (G/A). It was not possible to check for familial transmission for the A306T or rs368389701 as DNA was only available from the proband. Many of the rare variants were more common in PDB cases compared with controls. Individually, these did not reach statistical significance, but when information was combined for all rare variants with an allele frequency of <1%, the results were highly significant (OR = 3.72; 95% CI = 2.38–5.82; *P* = 8.9 × 10^−10^). Haplotype analysis showed that almost all rare variant's alleles (*n* = 24; 96%) occurred on the rs10498635C–rs117068593C risk haplotype background.

### Expression of RIN3 in bone cells

**Figure 3.**
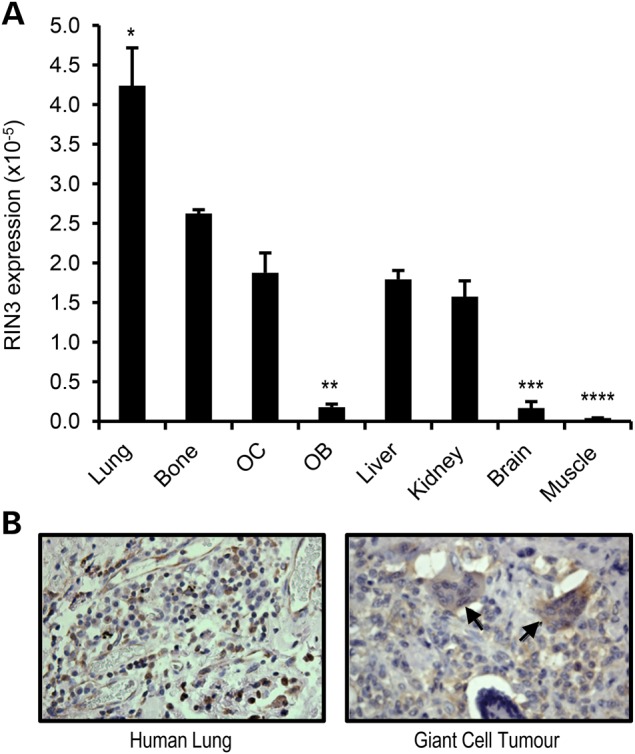
Expression of RIN3 in various tissues. (**A**) *RIN3* mRNA expression in the indicated mouse tissues and in cultured primary osteoclasts (OC) and primary calvarial osteoblasts (OB). RIN3 mRNA expression was normalized to 18 s rRNA and values are presented as mean ± SEM from three independent experiments. *P < 0.05 from all, **P < 0.05 from all except brain and muscle, ***P < 0.05 from all except OB and muscle, ****P < 0.05 from all except brain and OB. (**B**) Representative pictures of FFPE sections of lung (left) and giant cell tumour of bone (right) stained by immunohistochemistry for RIN3. Arrows pointing at stained osteoclasts. Magnification ×40.

Analysis of *RIN3* mRNA expression in mouse tissue showed that *RIN3* expression was highest in lung followed by bone tissue with the lowest expression level detected in brain, muscle and primary mouse osteoblast (Fig. 3A). *RIN3* mRNA expression in mouse osteoclast cultured from bone-marrow cells was about 10-fold higher than that observed in primary mouse osteoblasts (*P* < 0.001). RIN3 protein was also detected in human osteoclasts by immunohistochemistry of bone sections from a patient with giant cell tumour of bone (Fig. 3B).

### *In silico* analysis of variant pathogenicity

**Figure 4.**
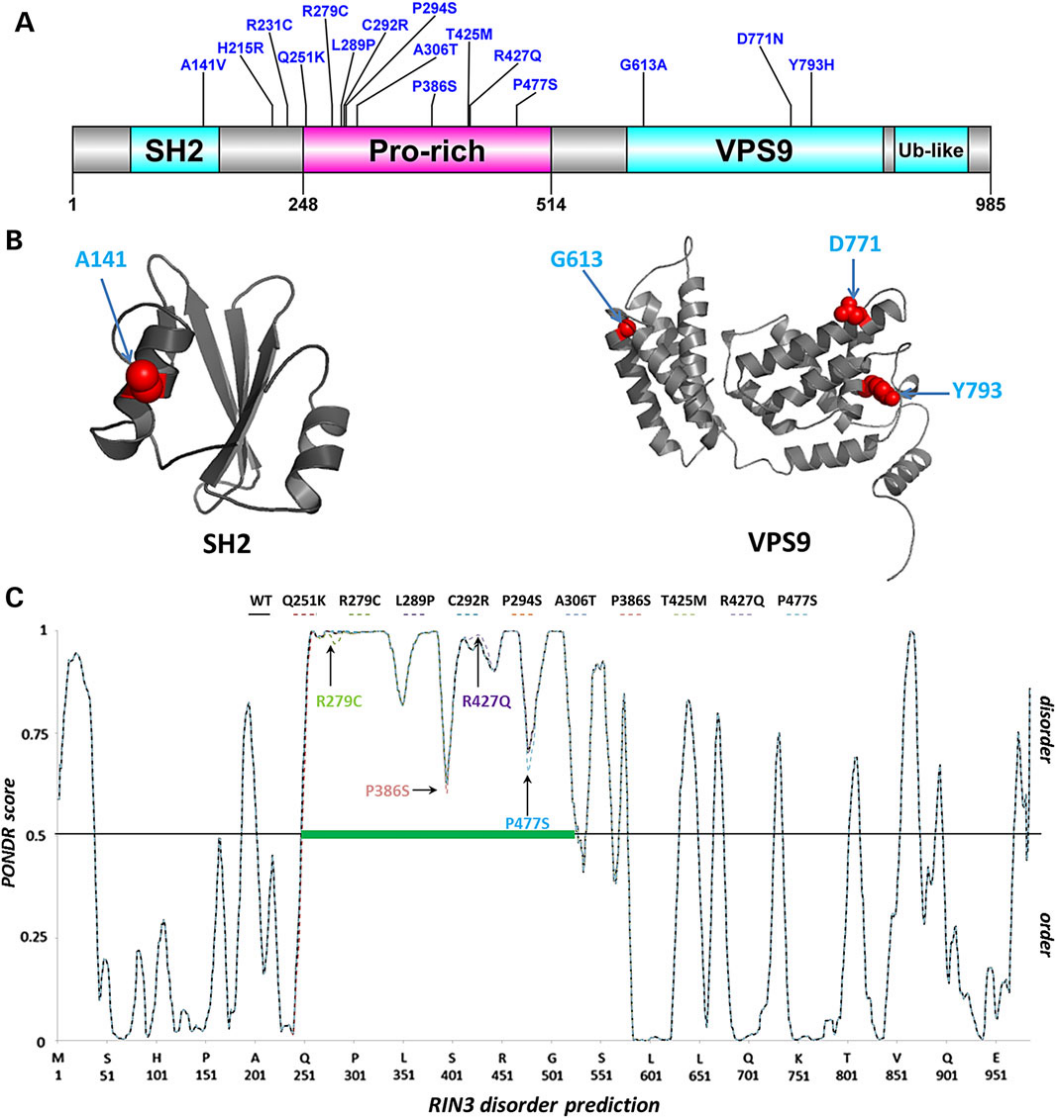
(**A**) The schematic of the RIN3 protein is presented with domains delineated and missense variants from this study mapped. (**B**) The 3-D homology models of the RIN3 SH2 and VPS9 domains are shown with location of variants indicated on structure. (**C**) The PONDR VL-XT disorder predictions (15) for wild-type and RIN3 variants are also presented drawn to scale with the schematic in the upper panel. The green bar denotes location of an extended region of predicted disorder that corresponds to the proline-rich region. Disorder predictions were carried out on full length wild-type and mutated protein sequences, generated by changing each of the variant residues individually *in silico* using PONDR. PONDR disorder scores <0.5 signifies predicted order, while ≥0.5 signifies predicted disorder. Only a subtle disordered-to-ordered structural transition is predicted for R279C, P386S and P477S, while an increase in disordered propensity is noticeable for R427Q. No change is discernible for the other variants. Only two out of the 16 variants (H215R and R231C) are located outside the modelled domains and disordered region, and hence were not assessed on this basis.

Information on conservation and likely functional significance for the identified missense variants as assessed by a suite of bioinformatics tools (see Materials and Methods) are shown in Table 1. The common variant p.R279C and four rare variants (p.A141V, p.R231C, p.P386S and p.Y793H) showed high functionality score (≥4) based on six different bioinformatics tools. The location of all missense variants in relation to RIN3 domains are shown schematically in Figure 4. The RIN family of proteins (RIN1, RIN2 and RIN3) share several common functional domains including an Src-homology 2 (SH2) domain, a proline-rich region, a vacuolar sorting protein 9 (VPS9) domain and an Ubiquitin-like Ras association domain (11,16). The conserved VPS9 domain is also present in the catalytic domains of guanine exchange factors Vps9p and Rabex-5 (16). The common protein coding variants and most of the rare variants that were associated with PDB (a total of 10 out of 16), cluster in the proline-rich region of RIN3 that encompass amino acids 248–514 (Fig. 4).

Application of meta-server consensus disorder predictor programs metaPrDOS (17) and MetaDisorder (18) (data not shown) along with PONDR VL-XT (15) suggest with high confidence that the proline-rich region corresponds to a region of extended intrinsic disorder (Fig. 4) in keeping with data that suggests that out of the 20 common amino acids, proline is the most disorder-promoting (19). Because currently available pathogenicity predictors are mainly structure- and/or conservation-based, their applicability in examining variants located in unstructured regions or regions of low sequence conservation in proteins (such as those variants located within the proline-rich disordered region of RIN3), are limited (20). The functional impact of these variants was therefore further assessed by their potential to alter disorder propensity, a known disease-mechanism, using PONDR, as was previously undertaken (20,21) (Fig. 4). The common variant p.R279C resides within a PXXP motif, a functional motif in several proteins that confers binding to a range of domains, including SH2 and SH3 (22); analysis of p.R279C showed a subtle tendency of disorder-to-order transition. We similarly analysed the other nine variants that reside within the proline-rich disordered region (Fig. 4). A noticeable increase in disordered propensity was found for R427Q, however, only a subtle disordered-to-ordered structural transition is predicted for P386S and P477S and no change is discernible for the other variants.

The location and predicted impact of missense variants that occur within structured domains was also assessed further by manually created three-dimensional (3-D) homology models. One missense variant (p.A141V) lies within the SH2 domain and three others (p.G613A, p.D771N and p.Y793H) reside within the VPS9 domain. While two of these mutations p.A141V (SH2) and p.D771N (VPS9) are solvent-exposed and thus are unlikely to impact on the stability of their domain structures, the possibility that they impact on protein–protein interaction remains. The p.G613A, however, present in the N-terminal helical bundle of VPS9, is not conserved even in mouse RIN3, and is likely to be a neutral substitution consistent with data from automated functional prediction tools (Functionality score = 1; Table 1). The p.Y793H variant that was suggested to be a ‘potentially damaging’ substitution with high probability on the other hand (Functionality score = 6; Table 1) is largely buried within the structurally and functionally important αV4 helix (23) in the VPS9 domain. Indeed, *in silico* mutagenesis of p.Y793H using FoldX (24,25) to assess its impact on structural stability produces a mean ΔΔG energy value ∼2 kcal/mol, suggesting that this mutant is destabilizing (24,26,27) where severely reduced structural stability is inferred by a ΔΔG > 1.6 kcal/mol. Similar mean ΔΔG energy values were obtained upon mutating the equivalent Tyrosine residues in each of the three templates used for modelling RIN3-VPS9, corroborating this result (Supplementary Material, Table S3).

## Discussion

The present study provides strong evidence that common and rare coding variants within the *RIN3* gene predispose to PDB. Association studies using imputation with the 1000 genomes panel as reference showed that rs10498635 is the only independent association signal in this region and suggested that the association was most probably driven by the coding variant p.R279C which was in strong LD with rs10498635. Haplotype analysis identified a common haplotype (rs10498635C–rs117068593C) associated with increased risk of PDB. Two other common variants (p.H215R and p.T425M) were detected in RIN3, but these showed no consistent association with the disease and they were not in LD with the GWAS top hit SNP rs10498635.

We also identified several rare protein coding variants which were strongly over-represented in PDB cases compared with controls (OR = 3.72; *P* = 8.9 × 10^−10^). Although the disease status was unknown for the 1000 genomes and NHLBI control cohorts, contamination of controls with undiagnosed cases is unlikely to influence the results substantially given that Paget's disease is a relatively uncommon condition occurring in about 1% or less of the European populations.

We found that *RIN3* was expressed in bone tissue and its expression level was ∼ 10-fold higher in osteoclasts compared with that observed in osteoblasts. RIN3 protein was also detected in osteoclasts from the curetted specimens obtained surgically from a patient with a giant cell tumour of bone. These data suggest that RIN3 may contribute to PDB susceptibility by affecting osteoclast function, consistent with the fact that PDB is considered a disease of abnormal osteoclast biology. However, further functional studies will be required to investigate the role of RIN3 in osteoclast differentiation.

Detailed *in silico* prediction identified two groups of variants; those located within structured domains of the protein (SH2 and VPS9) and those in the proline-rich disordered region that may be disease-causing. The common disease-associated variant p.R279C and most of the rare variants are located within the proline-rich disordered region of RIN3. Typically, disordered protein regions perform important cellular roles and bind to a range of protein partners owing to inherent structural plasticity (19). Interestingly, a recent study found that nearly 20–25% of disease mutations mapped to predicted disordered regions and 20% of disease mutations in these regions cause local disorder-to-order transitions (21). The p.R279C had a high functionality score as predicted by pathogenicity predictors (Table 1), whereas detailed *in silico* prediction analysis showed only a subtle order-enhancing effect (Fig. 4) consistent with the fact that this is a common variant with a modest effect size (OR = 0.64). Three other rare variants were predicted to have an impact on disorder propensity of this region of the protein and are thus likely to be solvent-exposed accessible to potential protein-interaction partners. Further functional analyses will be required to investigate the impact of variants located in the disordered region of RIN3.

The p.Y793H variant located in the VPS9 domain is predicted to be pathogenic by bioinformatics tools as well as by our homology modelling suggesting that this variant is highly likely to impair RIN3 function. Furthermore, functional studies have shown that mutations affecting the VPS9 domain of RIN3 impairs its GEF activity for Rab31 (12) consistent with the bioinformatics prediction.

It is interesting to note that the less common rs117068593T allele (encoding for cysteine at position 279) was associated with reduced risk of PDB, whereas the rare variants, when combined, were associated with increased risk of the disease. One explanation for this discrepancy comes from haplotype analysis which showed that almost all rare variant's alleles occurred on the risk rs117068593C haplotype background suggesting that the risk rs117068593C allele could be acting as a marker for rare variants. However, despite its weak predicted functional effect, it is possible for the rs117068593T allele to have a functional impact on the protein leading to protective effect on the disease. The pathogenicity predictors do not discriminate between mutations that confer loss of function, gain of function, or protective effects; they merely suggest a two-state prediction where a mutation is tagged as either ‘benign/neutral’ or ‘deleterious/pathogenic’. Furthermore, it has been shown that different mutations may have opposing impact on the protein function depending on their location and on the nature of the amino acid change involved. For example, certain missense mutations in *LRP5* gene cause high bone mass (28) and other conditions with increasing bone density (29), whereas other missense mutations in the same gene cause osteoporosis pseudoglioma syndrome (30).

While our *in silico* studies also revealed that some of the rare variants were predicted to be benign this does not exclude the possibility that they may be functionally important. A notable example of this in PDB is the case of the disease-causing p.P392L mutation in SQSTM1 which is predicted to be benign by bioinformatics tools but impairs the ubiquitin binding function of SQSTM1 (31).

More research will now be required to evaluate the role that RIN3 plays in bone metabolism and in the pathogenesis of PDB. The most extensively studied member of the RIN family is RIN1 which is known to affect signalling and stability of the EGF-receptor and other receptor tyrosine kinases (16,32–34). It is conceivable that RIN3 could act in a similar way to regulate signalling in osteoclasts but further studies will need to be done to investigate this hypothesis.

In conclusion, our study provides strong evidence that *RIN3* is the predisposing gene for PDB at the 14q32 locus and raises the possibility that RIN3 may play a previously unsuspected role in regulating bone metabolism.

## Materials and Methods

### Study population

Targeted sequencing of the entire *RIN3* gene was performed on leucocyte DNA from 121 patients with PDB and 49 controls. The PDB group comprised 95 cases who had participated in the PRISM study (35) and 26 familial cases in which one affected subject was selected from each family. The control group was selected from the PRISM control cohort (*n* = 40) and unaffected members of familial cases (*n* = 9). Sanger sequencing was performed on a group of subjects comprising 101 cases with PDB who had participated in the ZIPP study and 24 additional cases selected from the PRISM study. Both groups of patients were selected on the basis that they did not carry mutations in the *SQSTM1* gene and had an age at diagnosis of PDB of less than 65 years. All subjects used for DNA sequencing were of European descent and for the majority of cases (60%) genome-wide SNP data was available and ethnicity was confirmed by multidimensional scaling analysis (Supplementary Material, Fig. S1A). For the remaining subjects, ethnicity was determined by detailed questionnaire.

### Imputation

The imputation analysis was performed in 741 PDB patients and 2699 controls who had participated in previous genome-wide association studies in PDB as previously described (9). All subjects were of European descent as determined by multidimensional scaling analysis of genome-wide SNP data (see Supplementary Material, Fig. S1B). Genotype imputation across the 14q32 locus was performed using MaCH (36), and the 1000 Genome European-phased haplotype data (from phase I version 3) were used as a reference. We excluded SNPs with minor allele frequency of <0.01 and those with poor imputation quality based on the estimated correlation between imputed and true genotypes (*r*^2^ < 0.3). Imputed SNPs were tested for association using ProbABEL software (37) implementing a logistic regression model in which the allelic dosage of the imputed SNP was used to adjust for uncertainty in imputed genotypes. Regional association plots were generated using the LocusZoom tool (38).

### Sequencing

Sequencing of *RIN3* was performed using next-generation sequencing approach. A 210 kb region containing the entire *RIN3* gene and an ∼20 kb flanking region on either side was captured using the HaloPlex target enrichment kit from Agilent and following the manufacturer guidelines (Agilent, USA). Libraries were prepared and labelled with barcodes for sequencing on the Illumina HiSeq2000 platform. Sequence reads (Fastq) were mapped to the reference human genome (hg19/b37) using the Burrows-Wheeler Aligner (BWA) package (39). Duplicate reads were removed using Picard version 1.89 (http://picard.sourceforge.net) and Genome Analysis Toolkit ver 1.6 (GATK; http://www.broadinstitute.org/gsa/wiki/index.php/Home_Page) was used for local re-alignment of the mapped reads around potential indel sites. Base quality (Phred Scale) scores were recalibrated using GATK and calling of SNPs and indel variants was performed using the GATK unified genotyper. The mean target coverage was ×47, and 98.45% of aligned bases had quality scores >20. We excluded SNVs with quality score <20, those with coverage <×5 and indel variants.

The Sanger sequencing was performed on the promoter, coding exons and intron–exon boundaries of *RIN3* according to standard techniques using primer sequences described in Supplementary Material, Table S4. All primers were tagged with M13 sequences (GTAGCGCGACGGCCAGT on the 5′ extremity of the forward primers and CAGGGCGCAGCGATGAC on the 3′ extremity of the reverse primers). For standard PCR reactions, we used CM-102A Ready Mix Custom PCR Master mix (Thermos scientific) and primers at a final concentration of 0.83 µm. The melting temperature was 94°C for 1 min, annealing 58°C for 1 min and extension 72°C for 1 min for 30 cycles, preceded by an initial denaturation step of 94°C for 4 min, and final extension step of 72°C for 10 min. For reactions across CG-rich regions, a similar protocol was used except that we used Qiagen multiplex PCR master mix and Quaigen Q buffer. Here, the annealing temperature was 56°C, the number of cycles were 35, and the initial denaturation step was extended to 15 min. Following PCR, the reaction products were cleaned using the Agencourt CleanSEQ kit (Beckman Coulter) prior to sequencing and the sequencing reactions were performed using big dye version 3.1 reagents with M13 tagged forward and reverse primers. The sequencing reactions were analysed using the ABI 3130 and 3730 sequencers (Applied Biosystems). The sequencing results were analysed by the Mutation surveyor® V3.30 software (Softgenetics, State college, PA, USA) and compared with the reference sequences using Alamut V2.1 software (Interactive BioSoftware, Rouen, France). The primer sequences and reaction conditions used are provided in Supplementary Material, Table S4. As part of quality control measures, 15 samples were sequenced using both platforms and the genotype concordance was >99.5%.

### RIN3 mRNA expression and immunohistochemistry

Total RNA was isolated from adult mouse tissue/cultured primary cells and complementary DNA was generated by RT–PCR using the qScript cDNA SuperMix kit (QuantaBioscience) following the manufacturer's instructions. Expression of *RIN3* mRNA was measured by quantitative real-time PCR using Roche universal library probe system (Roche). A FAM-labelled probe (universal library probe 13: AGGCAGAG) was used to detect *RIN3* transcripts along with the following primer pair: forward, 5′GCCGGTCCTATTCCAGATG3′ and reverse, 5′AAGAACTGAGCCTTCCAGGTA3′. Real-time PCR was performed using SensiFAST Probe kit (Bioline) on a Chromo 4TM thermocycler (M J Research). *RIN3* expression levels were normalized to 18S rRNA which was determined using the VIC-labelled probe-primer combination from Applied Biosystems. Primary osteoblasts were isolated from the calvarial bones of 2-day-old mice by sequential collagenase/EDTA digestion and cultured in osteogenic medium (αMEM supplemented with 50 μg/ml vitamin C and 3 mm β-glycerol phosphate) for 21 days. To generate osteoclasts, bone-marrow cells were isolated from the long bones of 4-month-old mice and cultured for 48 h in the presence of M-CSF (100 ng/ml). Adherent cells were then stimulated with M-CSF (25 ng/ml) and RANKL (100 ng/ml) for 4 days until osteoclasts were formed. Osteoclasts were confirmed by tartrate-resistant acid phosphatase (TRAP) staining using a standard protocol. RIN3 expression in formalin-fixed paraffin-embedded (FFPE) human tissue sections was assessed by immunohistochemistry using RIN3 antibody (Sigma, cat number HPA039836) following a standard protocol. Sections of normal lung tissue from a patient with lung carcinoma and sections from a non-Pagetic case of giant cell tumour of bone were used with ethical approval.

### Data analysis

Association analysis of common SNVs was performed using χ^2^ allelic test and for rare SNVs we used Fisher's exact test. Burden test was performed on all SNPs with MAF < 1% (in our cases and/or controls) using Fisher exact test. Conditional analysis was performed using logistic regression implemented in PLINK v1.07 (40). Haplotype analysis was performed using Haploview (41). *RIN3* expression data were analysed using a two-tailed *t*-test.

### Functional annotation and assessing the impact of missense variants on structure

The regulatory regions within the area of interest were annotated using the Ensembl (www.ensembl.org) and ENCODE databases (www.genome.gov/encode) to predict their functional significance, that included assessment of transcription factor binding sites, splice-site loss or gain and known microRNA binding sites. The functional effects of protein coding variants was assessed using the following bioinformatics pathogenicity predictors: SIFT (42), PolyPhen-2 (43), Condel (44), MutationTaster (45), GERP conservation (46) and Grantham scores (47). A composite functionality score was devised in which each variant was assigned a score of one point for each assessment tool if it met the following criteria: predicted damaging by SIFT; predicted possibly or probably damaging by PolyPhen-2; predicted deleterious by Condel; predicted disease causing by MutationTaster, had a GERP score >2.0, or had a Grantham score >50. The functionality score ranges from 0 (low functional probability) to 6 (high functional probability).

Homology modelling of the SH2 and VPS9 domains of RIN3 was undertaken using Modeller 9v12 (48). FoldX (24,25) was used to estimate the free energy difference [stability change, expressed as delta delta Gibbs free energy (ΔΔG)] upon mutagenesis from wild-type. Meta-server consensus disorder predictor programs metaPrDOS (17) and MetaDisorder (18) were used to undertake disorder predictions on the full-length protein. PONDR VL-XT disorder predictions (www.molecularkinetics.com) (15) were carried out on full-length wild-type and each mutated protein sequence, generated by changing each of the variant residues individually.

## Supplementary Material

Supplementary Material is available at *HMG* online.

## Funding

This work was supported by a consolidator grant from European Research Council to O.M.E.A. (311723-GENEPAD) and by grants from Arthritis Research UK (19799 and 19520) to S.H.R. and O.M.E.A. This study makes use of clinical samples collected from the PRISM study funded by grants from Arthritis Research UK (18304) and the ZIPP study by grants from Arthritis Research UK (18163) and the Medical Research Council (09-800-05). Funding to pay the Open Access publication charges for this article was provided by the European Research Council.

## Supplementary Material

Supplementary Data
