# Supplementary material for: Targeted sequencing of the Paget's disease associated 14q32 locus identifies several missense coding variants in RIN3 that predispose to Paget's disease of bone
Source: Hum Mol Genet. 2015 Feb 20;24(11):3286–95. doi: 10.1093/hmg/ddv068 (PMC4424954; doi:10.1093/hmg/ddv068)
Supplement: Supplementary Data [file supp_24_11_3286__index.html]

Targeted sequencing of the Paget's disease associated 14q32 locus identifies several missense coding variants in RIN3 that predispose to Paget's disease of bone — Targeted sequencing of the Paget's disease associated 14q32 locus identifies several missense coding variants in RIN3 that predispose to Paget's disease of bone — Targeted sequencing of the Paget's disease associated 14q32 locus identifies several missense coding variants in RIN3 that predispose to Paget's disease of bone — Supplementary Data 

# Targeted sequencing of the Paget's disease associated 14q32 locus identifies several missense coding variants in *RIN3* that predispose to Paget's disease of bone

## Supplementary Data

Supplementary Data

**Files in this Data Supplement:**

- Supplementary Data - Pdf file
