# Supplementary material for: Targeted sequencing of the Paget's disease associated 14q32 locus identifies several missense coding variants in RIN3 that predispose to Paget's disease of bone
Source: Hum Mol Genet. 2015 Feb 20;24(11):3286–95. doi: 10.1093/hmg/ddv068 (PMC4424954; doi:10.1093/hmg/ddv068)
Supplement: Supplementary Data [file supp_ddv068_ddv068supp.pdf]

# Targeted sequencing of the Paget's disease associated 14q32 locus identifies several missense coding variants in *RIN3* that predispose to Paget's disease of bone


**Maheva Vallet[1], Dinesh C. Soares[2], Sachin Wani[1], Antonia Sophocleous[1], Jon Warner[3], Donald M. Salter[1], Stuart H. Ralston[1], Omar M.E. Albagha[1,*]**

[1]Rheumatology and Bone Disease Section, Centre for Genomic & Experimental Medicine, Institute of Genetics and Molecular Medicine, Western General Hospital, University of Edinburgh, Edinburgh, EH4 2XU, UK

[2]MRC Human Genetics Unit and Centre for Genomic & Experimental Medicine, Institute of Genetics and Molecular Medicine, Western General Hospital, University of Edinburgh, Edinburgh, EH4 2XU, UK

[3]South east Scotland Clinical Genetics Service, Centre for Genomic & Experimental Medicine, Institute of Genetics and Molecular Medicine, Western General Hospital University of Edinburgh, Edinburgh, EH4 2XU, UK

[*]Corresponding Author: Dr Omar M E Albagha, Centre for Genomic & Experimental Medicine, Institute of Genetics and Molecular Medicine, Western General Hospital University of Edinburgh, Edinburgh, EH4 2XU, UK; Fax +44 131 6511085, Telephone +44 1316511022, Email: Omar.albagha@igmm.ed.ac.uk

**Supplementary Table 1.** Missense and possible regulatory variants identified from deep sequencing of the entire *RIN3* gene region.

| Variant ID | Position (hg19) | Ref Allele | Sample Allele | Gene Region | Protein Variant | AF Cases[1] (%) | AF Controls[1] (%) | P-value[2] | AF 1000G[3] | AF NHLBI[4] | Functional Prediction[5] |
|---|---|---|---|---|---|---|---|---|---|---|---|
| N/A | 92979351 | A | G | Promoter | -- | 1/242 (0.4) | 0/98 (0.0) | 0.71 | -- | -- | NFE2L1/MafG |
| rs368389701 | 92980256 | C | A | 5'UTR | -- | 1/242 (0.4) | 1/98( 1.0) | 0.41 | -- | -- | TFBS (TAF1; POLR2A) |
| N/A | 93081806 | C | T | Exon 4 | p.A141V | 1/242 (0.4) | 0/98 (0.0) | 0.71 | -- | -- | 5 |
| rs3829947 | 93118038 | A | G | Exon 6 | p.H215R | 129/242 (53.3) | 60/98 (61.2) | 0.18 | 56.3 | 57.0 | 0 |
| rs117068593 | 93118229 | C | T | Exon 6 | p.R279C | 25/242 (10.3) | 24/98 (24.5) | $7.6 \times 10^{-4}$ | 17.0 | 19.6 | 4 |
| N/A | 93118260 | T | C | Exon 6 | p.L289P | 1/242 (0.4) | 0/98 (0.0) | 0.71 | -- | -- | 1 |
| N/A | 93118268 | T | C | Exon 6 | p.C292R | 1/242 (0.4) | 0/98 (0.0) | 0.71 | -- | -- | 2 |
| N/A | 93118310 | G | A | Exon 6 | p.A306T | 1/242 (0.4) | 0/98 (0.0) | 0.71 | -- | -- | 0 |
| rs3742717 | 93118668 | C | T | Exon 6 | p.T425M | 39/242 (16.1) | 15/98 (15.3) | 0.85 | 21.8 | 16.9 | 1 |
| rs12434929 | 93119232 | G | C | Exon 6 | p.G613A | 1/242 (0.4) | 0/98 (0.0) | 0.71 | 0.92 | 0.66 | 1 |
| rs145292991 | 93125790 | G | A | Exon 7 | p.D771N | 1/242 (0.4) | 0/98 (0.0) | 0.71 | 0.00 | 0.02 | 3 |
| rs147042536 | 93142861 | T | C | Exon 8 | p.Y793H | 1/242 (0.4) | 0/98 (0.0) | 0.71 | 0.66 | 0.63 | 6 |

1. Allele frequency (AF) shown as number of alleles observed / total number of alleles
2. Obtained from association testing of sequenced cases (n=121) and controls (n=49).
3. Allele frequency in European subjects from 1000 Genomes (n=379)
4. Allele frequency in European-American subjects in NHLBI dataset (n=4300)
5. For missense variants, as assessed by SIFT, PolyPhen-2, Condel, MutationTaster, GERP conservation score, and Grantham score (see methods).

**Supplementary Table 2**. Missense and possible regulatory variants identified from Sanger sequencing of *RIN3* promoter and coding region.

| Variant ID | Position (hg19) | Ref Allele | Sample Allele | Gene Region | Protein Variant | AF Cases[1] (%) | AF 1000G[2] (%) | AF NHLBI[3] (%) | Functional Prediction[4] |
|---|---|---|---|---|---|---|---|---|---|
| N/A | 92979351 | A | G | Promoter | -- | 1/250 (0.4) | -- | -- | NFE2L1/MafG |
| rs3829947 | 93118038 | A | G | Exon 6 | p.H215R | 128/250 (51.2) | 56.3 | 57.0 | 0 |
| N/A | 93118085 | C | T | Exon 6 | p.R231C | 1/250 (0.4) | -- | -- | 4 |
| rs147329151 | 93118145 | C | A | Exon 6 | p.Q251K | 2/250 (0.8) | -- | 0.02 | 1 |
| rs117068593 | 93118229 | C | T | Exon 6 | p.R279C | 29/250 (11.6) | 17.0 | 19.6 | 4 |
| N/A | 93118274 | C | T | Exon 6 | p.P294S | 1/250 (0.4) | -- | -- | 1 |
| rs201271121 | 93118550 | C | T | Exon 6 | p.P386S | 1/250 (0.4) | -- | -- | 4 |
| rs3742717 | 93118668 | C | T | Exon 6 | p.T425M | 38/250 (15.2) | 21.8 | 16.9 | 1 |
| rs74074811 | 93118674 | G | A | Exon 6 | p.R427Q | 2/250 (0.8) | -- | 0.12 | 0 |
| rs74074812 | 93118823 | C | T | Exon 6 | p.P477S | 1/250 (0.4) | -- | 0.02 | 3 |
| rs12434929 | 93119232 | G | C | Exon 6 | p.G613A | 3/250 (1.2) | 0.92 | 0.66 | 1 |
| rs147042536 | 93142861 | T | C | Exon 8 | p.Y793H | 4/250 (1.6) | 0.66 | 0.63 | 6 |

1. Allele frequency (AF) shown as number of alleles observed / total number of alleles
2. Allele frequency in European subjects from 1000 Genomes (n=379)
3. Allele frequency in European-American subjects in NHLBI dataset (n=4300)
4. For missense variants, as assessed by SIFT, PolyPhen-2, Condel, MutationTaster, GERP conservation score, and Grantham score (see methods).

**Supplementary Table 3**

| Mutation (Protein) | Stability energy calculation on mutant VPS9 domain structure (Mean ΔΔG) |
|---|---|
| Y793H in human RIN3-VPS9 domain homology model | 2.02 kcal/mol |
| Y193H in *Arabidopsis* VPS9A domain (2.08 Å crystal structure; PDB ID: 2EFE_A) | 2.32 kcal/mol |
| Y321H in human RABX5 domain (2.1 Å crystal structure; PDB ID: 2OT3_A) | 2.06 kcal/mol |
| Y321H in human RABX5 domain (2.35 Å crystal structure; PDB ID: 1TXU_A) | 2.91 kcal/mol |

FoldX stability calculations for equivalent conserved residues in the three template crystal structures used to build RIN3-VPS9 3-D model (> 1.6 kcal/mol is considered destabilising).

**Supplementary Table 4. Primer sequences for PCR and Sanger sequencing**

| | Forward | Reverse | Type of PCR |
|---|---|---|---|
| RIN3_PROM_A | TAATTCAGCCTCTGGAGGAG | GTACTGGCACATGCTACACC | Universal |
| RIN3_PROM_B | ATCACTAGAGTATTTTCTGTCTCT | ATCTGAGCATACACGAAAAGTC | Normal |
| RIN3_PROM_C | GGCCATATGCTTAACTTGACG | TGCTTTGTTATTAAACCTCCTTCA | Normal |
| RIN3_PROM_D | TAGAGACATTTCTGGTCGTTAC | CATCAAGCCTGGCATCTAATT | Normal |
| RIN3_PROM_E | AACACCCAGGCACTTAGAAGA | GGAGAGGGAAACTTAGAGAAAA | GC Rich |
| RIN3_PROM_F | AAGGCTGTGGCTCCGAGT | CCTTCTACTTCCTGTATCGG | GC Rich |
| RIN3_EX01 | TGACAAAAAAACCCTTGACCAC | AAAGTTTAGCCAACATCGGGT | Normal |
| RIN3_EX02 | ATAAGTAAGCGTGGCTGAATG | AAAGGGAAATAAACATGCAGTCAT | Normal |
| RIN3_EX03 | CTCATCATTTCAGGAACCTTC | AATGTGGCCATGAGAATGCAAA | Normal |
| RIN3_EX04 | TAATCTCCTGAAATCTCAATGGA | AAGCCTCAGAGCCAACACAT | Normal |
| RIN3_EX05 | AACCAAGGAGAAGCAGTGAC | GAAACTGGACAATCTCTCTATC | Normal |
| RIN3_EX06_A | AGCACAGCAACACCTAGTCC | AGTCTCTCGCAGGTCATCAT | GC Rich |
| RIN3_EX06_B | CTCTTGGAAATTGCCCTGCA | ACTTTGGTCTTCTAAGGACAC | Normal |
| RIN3_EX06_C | GGAAGCGATGAAGCCAGG | TCTGGAGAGCTCTGGGAAT | Normal |
| RIN3_EX06_D | AGCTCTGCACACAGGCGA | CTGCAGCAGGTAGCTCTTG | Normal |
| RIN3_EX06_E | ACTTTGGCAGCCTGGTGC | ATGGCAGGGAGTAATTGGCA | Normal |
| RIN3_EX07_A | TTTCTCTTGAATAAACTGTGCTCT | GGTGAACTTCTGCAGGATCTT | Normal |
| RIN3_EX07_B | AAGGATGGTTCGCTGCAGC | AAGAGGGCCCAGGAATTACA | Normal |
| RIN3_EX08 | GTCCTCTCTGTCCTGAGAG | CTAATCTGTGAGCTCCTGCA | Normal |
| RIN3_EX09 | TTGAAGCAGGTGTTTGCAGAT | GTGGGGAGTCTCACTGCT | Normal |
| RIN3_EX10_A | ACTCGCAGACAGCTTGGC | GGCCGGTAGACAAAGTGG | GC Rich |
| RIN3_EX10_B | GGAGAAGTTCGCGGTGGA | TTGGACGAGCGTCATGTTATTT | GC Rich |
| RIN3_EX10_C | CTTCCTGTGAGGCCCTC | GCTCCTTCCTAGGCCAGT | GC Rich |
| RIN3_EX10_D | ATTCCCCATGAGTCCCCC | AATTCTCCCCACACGATGG | Normal |
| RIN3_EX10_E | CGGTTAAGAGACAGGCCTC | TGGTTCTGATCCTAAGCTGG | Normal |

**Supplementary Figure 1**

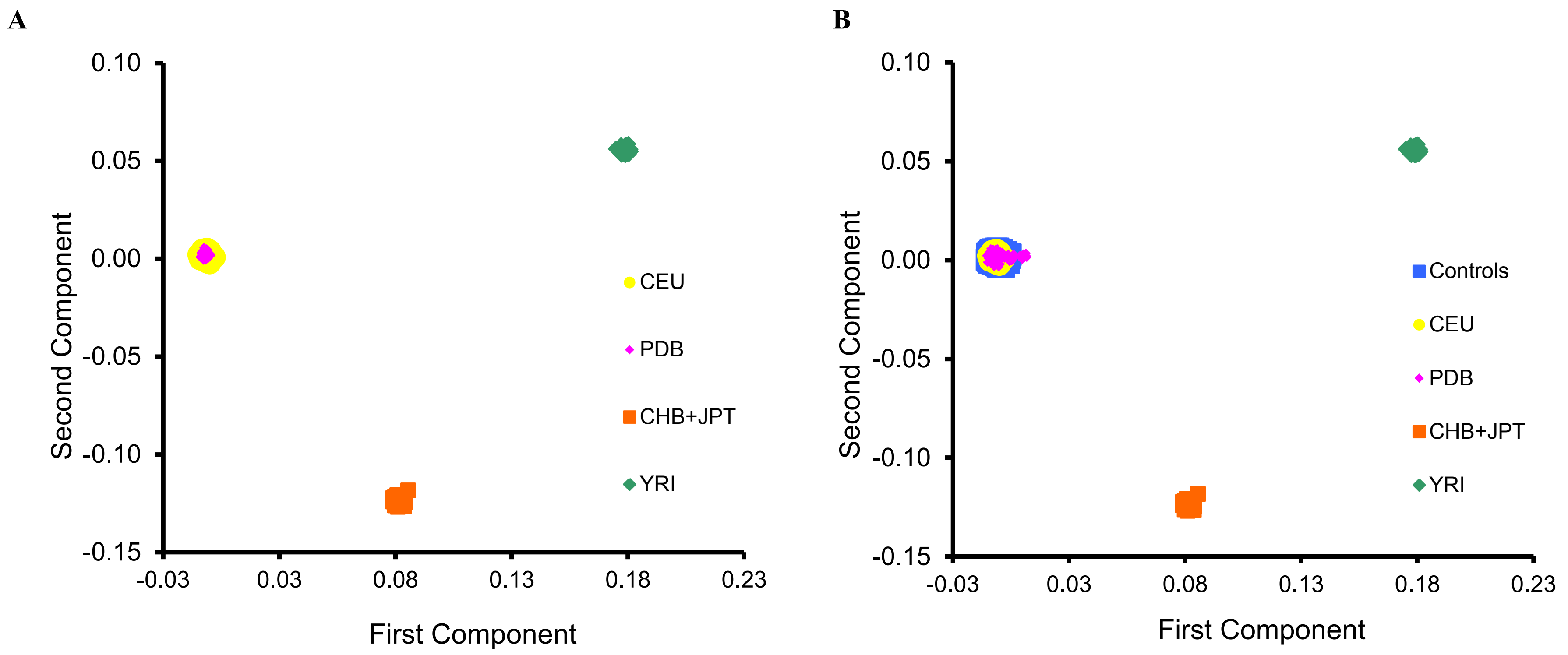


**Supplementary Figure 1**. Population ancestry for samples used in DNA sequencing (A) and for GWAS subjects (B). Multidimensional scaling analysis of genome wide SNP data for PDB cases (purple diamonds) and controls (blue squares) including HapMap project samples of European (CEU; yellow circles), Asian (CHB+JPT; orange squares), and African population (YRI; green diamonds).
